# Task Differences and Prosociality; Investigating Pet Dogs’ Prosocial Preferences in a Token Choice Paradigm

**DOI:** 10.1371/journal.pone.0167750

**Published:** 2016-12-21

**Authors:** Rachel Dale, Mylène Quervel-Chaumette, Ludwig Huber, Friederike Range, Sarah Marshall-Pescini

**Affiliations:** 1 Comparative Cognition, Messerli Research Institute, University of Veterinary Medicine, University of Vienna, 1 Veterinärplatz, Vienna, Austria; 2 Wolf Science Center, Ernstbrunn, Austria; Oregon Health and Science University, UNITED STATES

## Abstract

Prosociality has received increasing interest by non-human animal researchers since the initial discoveries that suggested it is not a uniquely human trait. However, thus far studies, even within the same species, have not garnered conclusive results. A prominent suggestion for this disparity is the effect methodology can have on prosocial responses in animals. We recently found evidence of prosociality in domestic dogs towards familiar conspecifics using a bar-pulling paradigm, in which a subject could pull a rope to deliver food to its partner. Therefore, the current study aimed to assess whether dogs would show a similar response in a different paradigm, based on the token exchange task paradigm frequently used with primates. In this task, dogs had the option to touch a token with their nose that delivered a reward to an adjacent receiver enclosure, which contained a familiar conspecific, a stranger or no dog at all. Crucially, we also included a social facilitation control condition, whereby the partner (stranger/familiar) was present but unable to access the food. We found that the familiarity effect remained consistent across tasks, with dogs of both the bar-pulling and token choice experiments providing more food to familiar partners than in a non-social control and providing less food to stranger partners than this same control. However, in contrast to our previous bar-pulling experiment, we could not exclude social facilitation as an underlying motive in the current task. We propose this difference in results between tasks may be related to increased task complexity in the token choice paradigm, making it harder for dogs to discriminate between the test and social facilitation conditions. Overall our findings suggest that subtle methodological changes can have an impact on prosocial behaviours in dogs and highlights the importance of controlling for social facilitation effects in such experiments.

## Introduction

Prosociality, defined as voluntary actions that benefit others [1], was considered by many to be a uniquely human trait until a spate of research over the last decade has found evidence in an ever growing number of non-human species. The most commonly employed experimental methodology for investigating these prosocial, or other-regarding, preferences is the prosocial choice test (PCT)- whereby individuals can typically choose between rewarding only themselves (selfish; 1/0) or rewarding themselves and their partners (prosocial; 1/1). These studies, however, have produced contradictory results, with findings seemingly being affected by the methodology, species and even specific individuals or dyads [2].

The bar-pulling PCT paradigm, in which animals can pull either a selfish or prosocial bar attached to baited shelves, has revealed prosocial responses in macaques and capuchins [3–6] but not in chimpanzees or tamarins [7–12]. Interestingly, when using a paradigm where subjects could exchange either a prosocial or selfish token, chimpanzees, capuchins and possibly parrots have been observed to be pro-social [13–16]. However, these prosocial findings have not been found by others, even when testing the same species (chimpanzees; [17]; capuchins; [18]). The issue with drawing conclusions from these disparate results, however, is that few of the studies are methodologically comparable. Differences include food visibility, extent of training, complexity of apparatus (e.g. tokens vs. bar-pull), and type of choice (e.g. number of options).

Drayton & Santos [19] did compare prosociality in capuchins that had completed two different PCT tasks and found prosociality in subjects to vary across paradigms. Indeed, comparing three individuals that completed both a touch screen task and a previous bar-pulling version of the PCT (see [5] for details on that paradigm), Drayton and Santos found that one individual was prosocial on only the bar-pull, another only on the touch screen and the third was not prosocial in either task. However, in the bar-pulling paradigm the animals’ understanding of the task was not assessed [5], and in the touch screen task the assessment showed a rather limited comprehension of the task contingencies [19]. Task understanding, therefore, may have played a role in the contrasting results of both of these paradigms. Also it should be noted that Drayton & Santos did not originally set out to directly compare the two paradigms.

On the other hand, House and colleagues [20] carried out one of the only direct comparisons of two prosocial tasks with the aim of disentangling some of the potential contributing factors to the variation in prosociality demonstrated by chimpanzees. One task was a variation on a PCT paradigm, whereby an actor could choose whether or not to pull a handle and obtain a food reward in an actor bin but could not obtain food in an out-of-reach receiver bin; any rewards in this bin were available to other group members. In the second task, subjects were presented with two sets of actor and receiver bins and could choose one of the handles to pull. In both tasks the number of food items and relative payoffs for both actor and receiver varied over trials, with some trials giving equal rewards to actor and recipient, some favouring the actor, and some favouring the recipient. Overall they found that in chimpanzees, the more complex the task (for example when the reward distribution meant more food items needed to be tracked) the less prosocial the individuals were, highlighting task complexity as a key confounding factor in prosociality studies.

Recently, we extended the research on prosociality to another taxon: Canids [21]. In this task, we used a simplified bar-pulling paradigm where domestic dogs could either pull a platform providing food to an adjacent enclosure or pull an empty one providing no food at all. We found that dogs preferentially pulled on the giving tray, i.e. the prosocial option, when a familiar conspecific partner was present in the adjacent receiver enclosure, compared to a stranger conspecific and control conditions where no partner could access the reward. Interestingly, they gave less to a stranger partner than in a control where the stranger was present but unable to access the reward and a control where no partner was present at all.

Crucially, we also controlled for social facilitation effects by including conditions where the partner was present but unable to access the food. Social facilitation is where the mere presence of a conspecific can increase, or inhibit, an individual’s motivational state, and in turn this can enhance or decrease its engagement in certain behaviours [22]. Although social facilitation has been controlled for in other paradigms (e.g. [23,24]), only one study so far has controlled for it in a PCT experiment [8] and here the chimpanzee’s level of prosociality did not differ from the test condition. Performance in our social facilitation controls did not differ from the non-social control, where no partner was present at all, but did differ from the test condition where the partner received the food, suggesting that the partner being able to access the food, rather than merely their presence, was driving the food delivery response of the dogs. In the current study, we investigated whether dogs would show similar responses in a token version of the PCT. In both studies, we used a simplified task similar to that previously employed with marmosets and rats [25–28]. In our tasks, donors could choose between rewarding a partner (giving; 0/1) and a control option, which gave no reward (0/0). We endeavoured to make the tasks as comparable as possible, such that in both the bar-pulling and the token tasks, we used the same conditions (familiar test (F.test), stranger test (S.test), familiar social facilitation control (F.SFC), stranger social facilitation control (S.SFC), non-social control (NSC)), testing rooms, food rewards and human experimenters (who were invisible after the first stages of training). In contrast to the bar-pull paradigm, where the dogs *pulled* a baited or non-baited shelf, in the token task, subjects learnt to *touch* (with their nose) a specific token which provided food, over another token which provided nothing. Having learnt this discrimination, they were given a free choice between ‘giving’ and ‘no reward’ tokens in the test and control conditions. Differently from the bar-pulling paradigm, in this task the food was not visible until after the choice had been made. Some authors have argued that food visibility makes the prosocial response less likely due to increased distraction by the visibility of the food, and thus reduced focus on the task at hand [13,29].

In line with our previous bar-pulling study, we predicted that a prosocial response towards the familiar partner would also emerge in the token task. Furthermore, based on the only studies assessing food visibility on prosociality [13,29], we predicted that the lack of visibility in the current task would enhance the prosocial response further. Hence, the prosocial response to the familiar partner should be even stronger in the token choice compared to the bar-pulling task, where the food was visible.

More specifically, for our first prediction to be met in the token paradigm we expected dogs i) to provide more food to a familiar dog than when it is absent from the testing room (non-social control) and when it is present but does not receive the food (social facilitation control), thus demonstrating prosociality, ii) to provide more to a familiar partner than a stranger, demonstrating consistency in familiarity effects, and iii) to show no difference in the number of trials the giving token was chosen when a partner was present in the room but unable to access the food (social facilitation) or not present (non-social control), ruling out social facilitation as an explanation for any prosocial behaviour.

Furthermore, when comparing the two paradigms, we expected dogs to show a significantly higher rate of ‘giving’ in the familiar test condition in the token paradigm, when the food is not visible at the time of choice, compared to the same condition in the bar-pulling paradigm, where the food was visible.

## Methods

### Ethical statement

All procedures were discussed and approved by the institutional ethics committee in accordance with Good Scientific Practice guidelines and national legislation (Ref. 06/05/97/2013).

### Subjects

Subjects were pet dogs from households of at least two dogs (N = 15, see S1 Table for details), whose owners voluntarily brought them on a weekly basis to the Clever Dog Lab of the Messerli Research Institute, University of Veterinary Medicine, Vienna. We also recruited 7 other dogs but they dropped out due to the owners ceasing to bring the dog (N = 5) or the dog not reaching training criteria (N = 2). None of our subjects acted as partners throughout the experiment and none had been subjects in the previous bar-pulling experiment or any other prosociality study. Familiar partners were dogs with whom the subject had shared the same household for at least one year and stranger partners were dogs of the same sex as the familiar partner, but from different households and with whom the subject had never previously interacted. Within a pair from the same household, the donor was selected randomly, but with the aim of keeping a balance in the number of males and females.

### General set-up

The general procedure of the training and test mimicked that of the bar-pulling study we previously conducted [21] in order to make a direct comparison.

**Fig 1:**
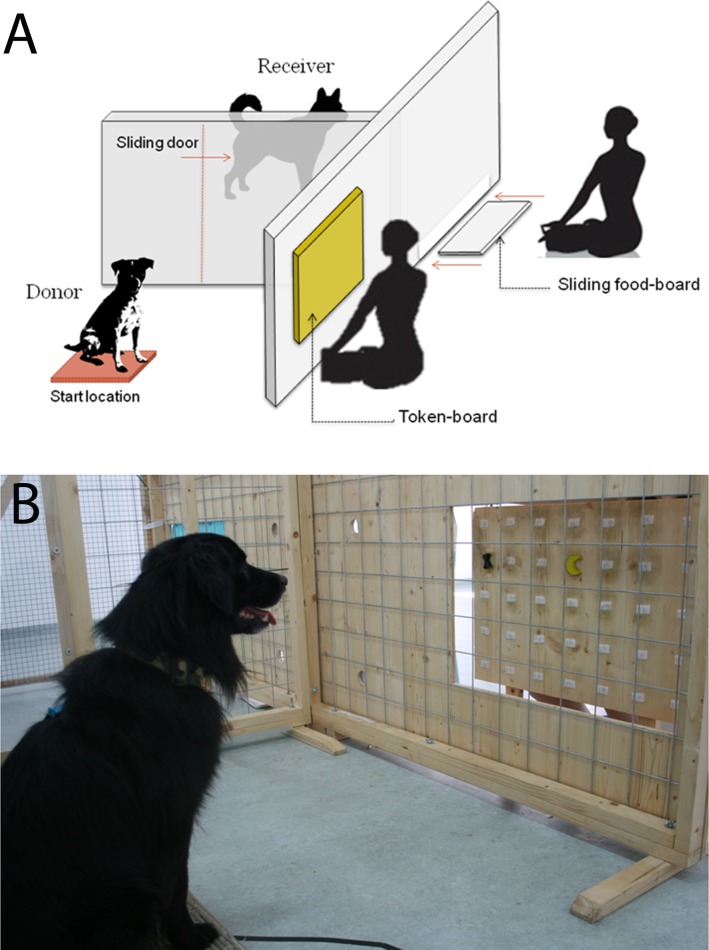
Experimental set-up of the token choice apparatus. A) The locations of the donor, receiver and apparatus. B) The token board from the donor dog’s perspective, the moment before the board is pushed forwards and a choice can be made.

The set-up consisted of two enclosures, donor and receiver, with a transparent sliding door between them that could be opened or closed (Fig 1A). The fronts of the enclosures were covered by a wooden board to make the experimenters invisible. The enclosure could otherwise be seen through, including between the donor and receiver. In the donor enclosure there was a hole in the wooden board for the token board to be placed during each trial. On this token board were 15 possible locations for wooden tokens to be attached using Velcro® (Fig 1B). On each trial, two tokens (one giving and one control) would be placed in semi-randomised locations on the board by the experimenter, such that a token never appeared in the same location more than two trials in a row and the tokens were never directly next to each other. Between each trial, the token board was removed and a curtain covered the hole to ensure the experimenters remained invisible. Before each trial, dogs were instructed to sit on a start location, a wooden board covered by a mat on the floor at the back of the enclosure, directly opposite the token board position.

When the ‘giving’ token was touched by the donor, a small tray containing a food reward (a piece of sausage) was slid under the wooden board into the receiver enclosure, adjacent to the fence separating the two enclosures (see Fig 1A). When the control token was touched the dogs were given a few seconds to explore the enclosure before the next trial was started. The experimenter could see which token the dog selected via a webcam placed next to the donor’s enclosure and connected to a laptop which was placed next to the experimenter.

### Training

**Fig 2:**
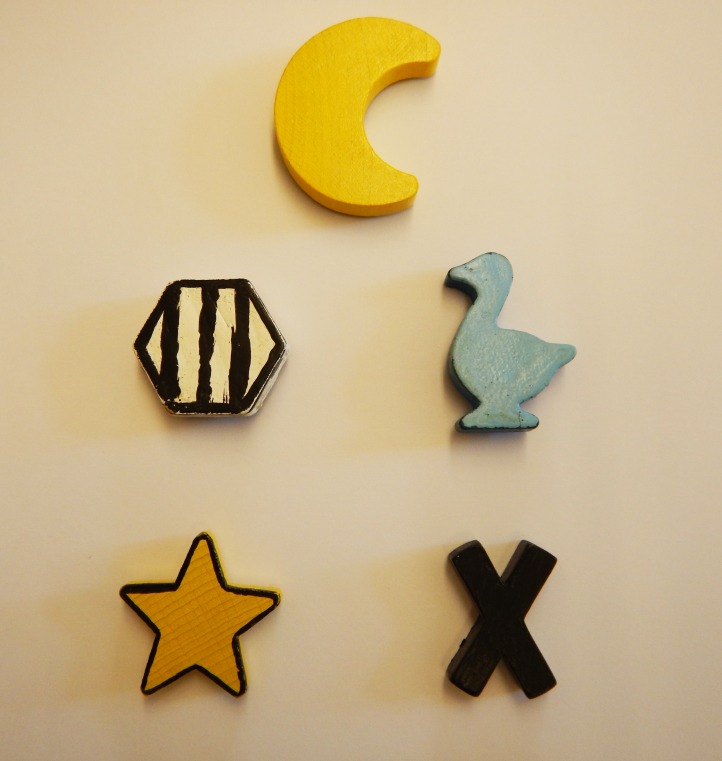
The five tokens used in the experiment. The yellow moon was the step 1 training token for all dogs. From the remaining four tokens, each dog was assigned two of these; one giving and one control.

Training was composed of two parts. In the first part, dogs were trained to sit on the ‘start location’ within the donor enclosure and received a reward for doing so. Separately, they were trained to touch a wooden training token (different from the tokens used in the test; Fig 2) in exchange for a food reward. Once these two behaviours were acquired separately, dogs moved on to the second step. From this stage on, dogs were no longer rewarded for sitting on the start location.

In the second step, subjects were asked to sit on the start location and stay there whilst the experimenter opened a curtain to reveal the board, held 3 cm away from the frame, with the tokens randomly located on it (Fig 1B). After three seconds, where the dogs could observe the tokens, the experimenter pushed the token board forward making the tokens available to the dog. At this stage, dogs could now choose between the two tokens. One was a ‘giving’ token, which, when touched, resulted in a reward being delivered to the adjacent enclosure, the other was a ‘control’ token, which gave nothing. Dogs were randomly assigned these tokens from a pool of four tokens (Fig 2). During this training the door between donor and receiver enclosures was open (Fig 1A), so if dogs selected the giving token, they could go into the receiver enclosure and take the food. If they did not choose the giving token, they were given 10 seconds to explore the enclosure before being asked to go back to the start location. Dogs visited the lab once or twice a week during training and completed no more than two training sessions (of 20 trials each) per visit. In order to proceed to the test, dogs had to successfully touch the giving token and retrieve the reward in the receiver enclosure in 17 trials out of 20. Dogs took an average of 7.36 sessions to complete this training step.

### Test and control conditions

Each donor participated in one session each of the two test and the three control conditions (S1 Movie). The conditions were run in a semi-randomised and counterbalanced order such that the two conditions involving a particular partner (test condition and social facilitation control of familiar or stranger dog) were run one after each other. Moreover, we took care that half of our subjects experienced the familiar conditions before the stranger conditions and, the other half started with the stranger conditions before the familiar.

Both test and control sessions comprised a maximum of 40 trials each and only one session per day was carried out. A session ended either if the donor did not touch a token on five consecutive trials or if donor dogs refused to move to the start location prior to the next trial, despite five consecutive requests from the experimenter. These requests consisted of calling the name of the dogs followed by the command “go sit” by the experimenter holding the token board.

### Motivation sessions

Because the test occurred under extinction conditions, motivation sessions were run between each condition (but on separate days) in order to ensure that the motivation to touch the tokens was similar between our various test and control conditions (see below). These sessions mimicked the final training sessions, such that the donor dog was alone and the sliding door between the two enclosures was open allowing the dog to retrieve the food in the receiver enclosure after touching the giving token. Dogs had to touch the giving token on 17 out of 20 trials (subjects took an average of 1.51 sessions between each test/control condition; probability of success on first session = 0.73, p < 0.001) before they were allowed to proceed to the next experimental session.

By using this method, we ensured the subjects returned to a baseline level of motivation to perform the task before entering the next test or control condition.

### Self-rewarding trials

**Fig 3:**
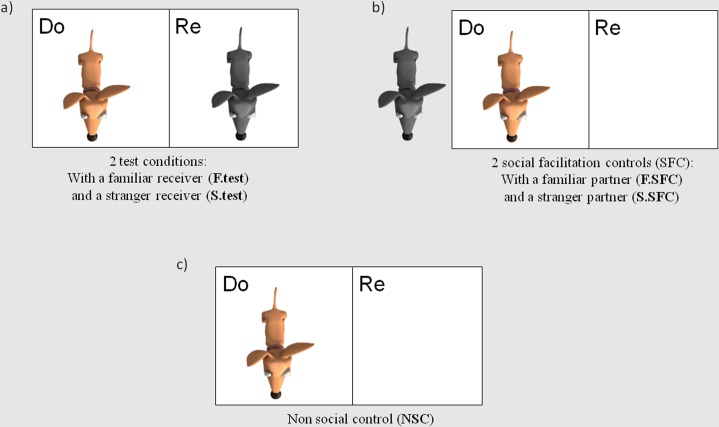
Location of the donor (Do) and receiver (Re) for each condition. Figure taken from Quervel-Chaumette et al [21].

At the end of each session (test and control) donor and partner (when present) remained in their original locations (Fig 3) and we conducted four “self-rewarding” trials where touching the training token (which always delivered food to the donor during training, Fig 2) now delivered food to the donor’s enclosure. These four trials ensured that dogs were still motivated to perform the task at the end of a session and that even when paired with a stranger, dogs were still comfortable enough to perform the trained action if they could obtain food for themselves. These trials ensured that the number of giving trials performed in the test conditions were a reflection of prosociality, rather than stress, distraction or motivation.

### Analyses

There were ultimately three measures to consider per session. Firstly, the total number of trials across conditions, before the subjects ended the session, including trials where they did not touch a token. Furthermore, if they decided to touch, they could either choose the giving or the control. Hence we also took into account the total number of touches regardless of token choice (i.e. including both giving and control) and the number of giving choices across conditions (i.e. touching the token that delivered food to the partner enclosure). Therefore, initial analyses investigated the potential correlations between each of these three dependent variables using a Pearson’s correlation test.

Having established a high correlation between these three measures (see below), we chose the number of giving choices (S1 Data) as the main dependent measure for the subsequent analyses and used likelihood ratio testing to assess the statistical influences of the various explanatory factors. In order to examine if the number of giving choices differed across sessions and conditions, *g*eneralized linear mixed models (GLMM), correcting for overdispersion by using the glmmADMB package and function in R version 3.2.2, were used with the donor’s identity as a random factor and the number of ‘giving touches’ as the response factor. The order of sessions (i.e. first to fifth, to check for order effects) and condition (familiar test, stranger test, familiar social facilitation control, stranger social facilitation control and non-social control) as well as the interaction between session and condition were included in the model as explanatory factors.

#### Behaviour coding

The video recordings of the test and control sessions were coded with Solomon Coder Beta 15.01.13 (Copyright András Péter, http://solomoncoder.com). Ten videos were not coded due to technical problems during recording. The behavioural categories and ethogram used were the same as those used in Quervel-Chaumette et al [21] (see S2 Table for details).

We created a category of ‘stress behaviours’ and within this coded all occurrences of scratching, yawning, lip-licking and attempts to leave the donor enclosure shown by the donor. The presence of agonistic behaviour (growling, snapping and threatening) by both the donor and receiver was coded as a binary variable (0 for none, 1 if it occurred in a session). Finally, in the test conditions we coded the frequency with which the partners (familiar and stranger) tried to reach the reward by, for example scratching at the fence/apparatus (see S2 Table for details of the ethogram). The behaviour coding was done by one author (RD) and twenty percent of the videos (N = 15) were coded by a second observer (MQC). Cronbach’s alpha coefficient was used to measure the level of agreement. For all behaviours the alpha was between 0.77 and 0.87, which corresponds to a high level of agreement [30].

Linear mixed models were run with the identity of the donor as a random effect, the frequency of “stress behaviours” as the response factor and session and condition as fixed factors. Due to overdispersion of this model, a glmPQL in the ‘nlme’ package was run to control for this.

Finally, a Pearson’s correlation test between the number of giving pulls and the frequency of attempts to reach for the food by the partner in the familiar and stranger tests was used to examine whether donors were more likely to provide food when the partner tried to reach the food.

## Results

### Number of trials, touches, and giving touches

Pearson’s correlation tests showed a high correlation between the three possible outcomes of a trial, in that if donor dogs chose to participate in a trial they normally touched a token (R = 0.89), and if they touched a token it was in most cases the giving token (R = 0.97). Therefore the number of giving choices was chosen as the dependent measure for further analyses as this quantifies the food delivered to the receiver enclosure and thus represents the best measure of other-regarding preferences.

### Effect of condition

**Fig 4:**
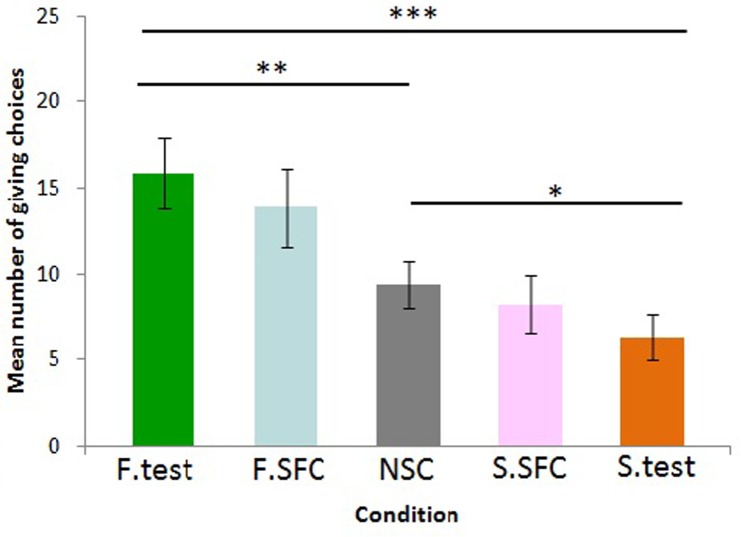
Mean number of giving choices by donors across conditions (*p<0.05, **p<0.01, ***p<0.001). F = familiar, S = stranger, SFC = social facilitation control, NSC = non-social control. Error bars represent one standard error of the mean.

The GLMM model revealed no interaction effect between condition and session on the response variable (χ^2^(4) = 3.23, p = 0.5), nor was there a main effect of session (χ^2^(1) = 3.12, p = 0.07). However, the condition influenced the number of giving trials performed by the donor (χ^2^(4) = 10.49, p = 0.03; see Fig 4). Specifically, when paired with a familiar receiver, donors gave more food than when the receiver was a stranger (F.test: mean = 15.9 s.e ± 2.1; S.test: mean = 6.3 s.e ± 1.4; GLMM: z = -4.48; p<0.001 after Bonferroni correction; Fig 4). Importantly, donors also touched the giving token more in the familiar test than in the non-social control where no partner was present at all (NSC: mean = 9.4 s.e ± 1.4; GLMM: z = 2.6; p<0.01). Interestingly, as in the bar-pulling study, the dogs selected the giving token more in the NSC than in the S.test, where a stranger received the food (GLMM; z = -1.88, p = 0.05). Furthermore, there was no difference between the NSC and either of the social facilitation controls (F.SFC: mean = 13.9 s.e ± 2.3; GLMM: z = 1.72, p>0.05; S.SFC: mean = 8.3 s.e ± 1.6; GLMM: z = -0.8, p>0.05). However, we could not rule out social facilitation entirely, with donor dogs not differing in their performance between F.test and F.SFC, where the familiar partner was present but unable to access the given food (GLMM: z = -0.92, p>0.05), nor between S.test and S.SFC (GLMM: z = -1.13, p>0.05).

### Self-rewarding trials

In the four self-rewarding trials at the end of each session, subjects (and partners when present) remained in their original location and dogs had to select the training token, which delivered food to their own enclosure. As with the bar-pull study [21], all subjects touched the training token in order to deliver food to themselves on 100% of “self-rewarding” trials.

### Behavioural measures

Firstly, neither condition nor session influenced the frequency of stress behaviours exhibited by the donors (condition: χ2 = 10.34; p = 0.17, session: χ2 = 0.44, p = 0.51). Only one instance of aggression occurred over all testing, therefore statistical comparisons across conditions were not run. This demonstrates that the set-up did not provoke aggressive interactions between unfamiliar dogs, nor did the receiver dogs eating the rewards provoke aggression. Finally, no significant correlation was found between reaching for the food by the partner and the number of giving trials (mean_reach food_ = 1.5 per session, r = 0.05, p = 0.78).

### Comparison with the bar-pulling paradigm

In order to investigate the effect of paradigm on prosociality in dogs, we statistically compared the results of the bar-pulling and token tasks (Table 1).

**Table 1:** comparison of the mean and standard error values in each condition for both the token and bar-pull paradigms

Condition	Mean		S.E	
	Bar-pull	Token	Bar-pull	Token
**F.test**	20.5	15.9	1.9	2.1
**S.test**	8.9	6.3	1.9	1.4
**NSC**	13.3	9.4	2.4	1.4
**F.SFC**	12.9	13.9	2.5	2.3
**S.SFC**	13.2	8.3	1.6	1.6

**Fig 5:**
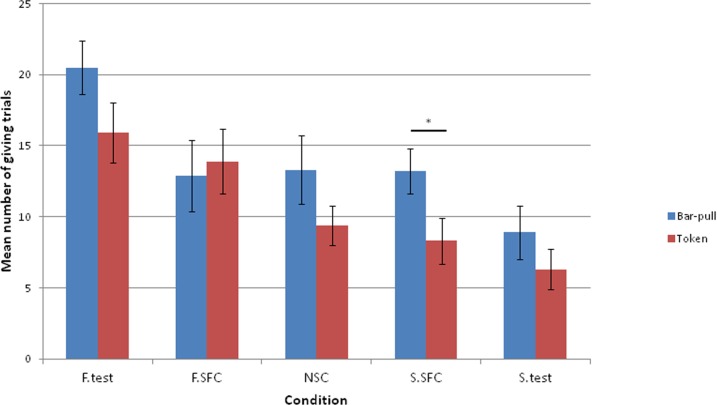
Mean number of giving choices in each condition for both the bar-pulling and token choice experiments.

When the data from the two tasks was combined, a GLMM revealed an interaction between condition and experiment (χ^2^(1) = 10.05, p = 0.04). Therefore we looked at whether there was an effect of experiment on each condition separately. We found that donors performed significantly fewer trials in token choice than the bar-pulling task only in the stranger social facilitation control (χ^2^(1) = 6.04, p = 0.01, Fig 5), in all other conditions there was no effect of paradigm (F.test; χ^2^(1) = 2.9, p = 0.09, S.test; χ^2^(1) = 0.73, p = 0.39, F.SFC; χ^2^(1) = 0.22, p = 0.64, NSC; χ^2^(1) = 1.37, p = 0.24).

## Discussion

In summary, results from the current task showed that when given the possibility of delivering food to a conspecific receiver, dogs were more likely to act in a prosocial manner when the conspecific was familiar than when it was a stranger. Importantly, in a control condition where no receiver was present to access the food, dogs delivered less food than when a familiar conspecific was present. Interestingly, dogs delivered less food when a stranger receiver could access the food than in the same control where no receiver was present at all. These results are in line with our first prediction that dogs would be prosocial to familiar partners and are consistent with our previous study where a bar-pulling apparatus was used instead [21]. However, this prediction was not fully supported since, unlike in the bar-pulling paradigm, in the present token choice paradigm, dogs did not differentiate between when the partner received the reward (test condition) and when it was present but could not reach the treat (social facilitation control condition), and hence a social facilitation effect could not be ruled out as a motivational factor (see below for a more in depth discussion of this issue).

Furthermore, our results also contradict our second prediction. Dogs were not more prosocial in this task, where the food was invisible, than in the bar-pulling where the food was visible during the choice phase of the task. These results are in contrast to previous suggestions from studies with primates that food visibility reduces prosociality [13, 29].

The primary question raised from these results is why dogs in the token test did not differentiate between the social facilitation control condition and the test condition. There are a number of potential explanations as to why this difference between the two tasks may have arisen. Firstly, it could be argued that our subjects did not understand the task contingencies here. However, when subjects could work for themselves during the trials at the end of each session, they were always willing to do so, suggesting that they were still motivated to work when they themselves received the reward. Additionally, the subjects did discriminate between the two test conditions and the non-social control, suggesting an understanding of the task set-up. Nonetheless, there was no difference between social facilitation controls and the test, despite a general understanding of the task, suggesting the dogs did not make the distinction between these two conditions, were not attentive to it, or that the mere presence of the partner was sufficient to drive the prosocial behaviour.

Indeed, it is possible that some form of emotional response (social facilitation), whereby no matter where the partner is, their mere presence elicited (for the familiar) or inhibited (when the stranger was present) the subject to act. This may additionally suggest that social facilitation may be an underlying mechanism of prosocial responses, which is in line with previous findings on the importance of social factors in helping behaviours in rats [28]. However, if this was the only feature at play in the responses of the dogs, we should have also found a difference between the social facilitation controls (stranger and familiar) and the non-social control. On the contrary, although we found no difference between the test and social facilitation controls, we also found no difference between the social facilitation controls and the non-social control, suggesting that just the presence of the partner in the room was not significantly increasing or decreasing the activity levels of the donors. Therefore, although social facilitation cannot be totally excluded, it also cannot be considered the main explanation for the dogs’ response in the test conditions. Furthermore, if social facilitation were the main phenomenon driving the dogs’ ‘giving’ behaviours we would have expected such an effect to also emerge in the bar-pulling paradigm, but this was not the case.

Consequently, the more likely explanation is that differences in methods and materials between the two set-ups may have made it harder for dogs in the token choice test to discriminate between the condition in which the partner was present and also receiving the food, vs. when the partner was present but had no access to it. The primary task difference was in the food visibility during the choice of the donor. In the current task the food was not visible during the choice phase, which some authors have argued makes the prosocial response more likely [13,29], since actors are not too distracted by visible food to focus on the task. However, our results do not appear to support this hypothesis in the current context. Indeed, when comparing the number of giving trials between the two tasks, it appeared that dogs were equally prosocial towards the partner in the familiar test. In fact, although not statistically significant, donors were even slightly more prosocial in the bar-pulling task, delivering on average 20.5 vs. 15.9 food items to their partner. Hence if at all, food visibility enhanced dogs’ prosocial response in this context, potentially because it helped animals keep track of the consequence of their actions more easily.

The invisibility of the food in the token choice experiment may have actually added to the attentional and cognitive demands of the task, as dogs had to keep out-of-sight food in mind, whereas in the bar-pulling task, being able to track the food from the moment it was placed on the tray to the mouth of the partner (or the empty enclosure), may have helped dogs to discriminate between the conditions in which the partner obtained the treat or not. Although sense of smell may have played a role in the dogs more so than in primates (on which this hypothesis was based), studies have shown that in choice tasks dogs do predominantly use their sight [31,32]. However, it cannot be ruled out that the use of the visual sense may be different in canids and primates in such tasks.

Furthermore, it could be argued that the token choice task is in general more cognitively demanding than the bar-pull paradigm. Indeed, relative to the bar-pulling task, in the token choice dogs not only had to learn an abstract association between a symbol and the food reward (as opposed to the direct connection between food and shelf in the bar-pull), but also had to track the location of the token in one of 15 possible locations on the board (compared to choosing between a top or bottom shelf in the bar-pulling). As such, it may be that dogs had to both remember and pay attention to more information in the token choice than in the bar-pull. The number of sessions required to reach the training criterion (selecting the giving option on 17/20 trials) supports the idea that the token choice task was more demanding than the bar-pulling task, with dogs needing a mean of 2.38 sessions in the bar-pull and 7.36 in the token choice. The reduced prosocial response in the relatively more complex token choice task is in line with findings by House et al [20] in chimpanzees, and also results from human children [29], that the more cognitively difficult the task, the less likely are subjects to exhibit prosocial behaviours.

The increase in cognitive demands may explain why dogs did not appear to clearly distinguish between social facilitation and test conditions. The more complex the task, the harder it was for animals to focus on whether the partner had access to the food or not.

To further our understanding of how the performance of the dogs differed between the tasks, we additionally directly compared the number of giving trials in each experiment. In fact, the only significant difference we did find was in the stranger social facilitation control, with dogs performing fewer giving trials in the token choice than in the bar-pull. Based on the current findings we cannot yet explain the reason for this. One interpretation could be that because in the token choice the dogs needed to be close to the stranger to touch the token board, as this apparatus was located on the outer edge of the enclosure near the ‘control position’ of the partners, donors were not comfortable to move close to them. The bar-pull apparatus, on the other hand, was located in the inner part of the enclosure, away from the stranger in this control. However, in the bar-pull dogs were still required to be close to the stranger in the control in order to complete a trial, as the starting location where dogs had to sit at the start of each trial was on the outer edge of the compartment. Additionally, the behavioural patterns were very similar between the two studies, namely that no differences in stress or aggressive behaviours were found across conditions. It is still possible though, that the dogs perceived a difference between working (token) and sitting (bar-pull) near to the stranger in terms of motivation or discomfort. However, at this stage this is simply a suggestion that requires further investigation.

What this difference between the two tasks in this condition does highlight however, is i) that dogs, and probably other animals, are sensitive to rather subtle methodological changes in prosociality studies and ii) the social facilitation control is an extremely important condition to include in order to disentangle any differences between test and non-social control conditions (see also [2]). Other than the current study and the previous bar-pull task, this control has only been used in one other PCT paradigm study to date and the authors also found that chimpanzees chose the prosocial option equally often when the partner was, and was not, able to access the food [8]. This suggests that social facilitation may also be playing a role in the prosocial responses found in other species. Furthermore, studies using other prosociality paradigms have found that social facilitation may sometimes explain helping responses in animals [24].

Based on the findings from both of our tasks it appears that multiple, but comparable, paradigms are needed to give us an insight into the specific conditions under which prosocial behaviour is shown. Importantly, the present comparative work demonstrates that the social facilitation control should be an essential condition included in future prosociality studies.

## Supporting Information

S1 Table Subject information (DOCX)

S2 Table Ethogram of behaviours used for video coding (DOCX)

S1 Data Supporting data. (XLSX)

S1 Movie Video showing examples of each test condition (AVI)
