# Supplementary material for: Task Differences and Prosociality; Investigating Pet Dogs’ Prosocial Preferences in a Token Choice Paradigm
Source: PLoS One. 2016 Dec 21;11(12):e0167750. doi: 10.1371/journal.pone.0167750 (PMC5176280; doi:10.1371/journal.pone.0167750)
Supplement: S1 Table — (DOCX) [file pone.0167750.s001.docx]

| Donor | Donor sex | Donor age | Donor breed | Number of step2 training sessions before testing | Familiar partner | Stranger partner | Partner sex |
| --- | --- | --- | --- | --- | --- | --- | --- |
| Teddy | M | 9 | Belgian shepherd | 14 | Lola | Hybie | F |
| Flamme | M | 7 | Pyrinean shepherd | 2 | Nessie | Lola | F |
| Joey | M | 8 | Mix | 2 | Flappi | Buck | M |
| Michel | M | 10 | Mix | 18 | Monty | Buck | M |
| Sokrates | M | 9 | Podenco Canario/Bardino | 15 | Ultimo | Buck | M |
| Quismo | M | 8 | Border collie | 5 | Luke | Buck | M |
| Luna | F | 3 | Husky | 6 | Tallie | Buck | M |
| Hybie | F | 6 | Mix | 5 | Tuukka | Rosie | F |
| Amy | F | 9 | Border collie | 2 | Baco | Mago | M |
| Emily | F | 3 | Bernese mountain dog | 2 | Aiko | Mago | M |
| Charlie | M | 7 | Bearded collie | 7 | Cookie | Michel | M |
| Zuri | F | 8 | Rhodesian ridgeback | 4 | Akin | Mago | M |
| Akyla | F | 2 | German shepherd | 10 | Nya | Lola | F |
| Emma | F | 2 | German shephard | 6 | Hanja | Lola | F |

S1 Table: Information for each subject of the token choice experiment.
