# Supplementary material for: Task Differences and Prosociality; Investigating Pet Dogs’ Prosocial Preferences in a Token Choice Paradigm
Source: PLoS One. 2016 Dec 21;11(12):e0167750. doi: 10.1371/journal.pone.0167750 (PMC5176280; doi:10.1371/journal.pone.0167750)
Supplement: S2 Table — (DOCX) [file pone.0167750.s002.docx]

| Behaviors |  | Definition |
| --- | --- | --- |
| Stress behaviors | Yawning | Opening the mouth wide and then closing it. |
|  | Lips-licking | To lick the lips |
|  | Attempt to leave enclosure | The body is orientated toward the exit. The nose is touching the end of the fence. Dogs may be pushing the fence with the head or pawing at it or simply standing in front of the exit. |
|  | Scratching | Scratching any part of the body |
| Agonistic behaviors | Threat | Subject orients towards another performing one or more of the following: staring at, curling of the lips, baring of the canines, raising the hackles, snarling, growling, and barking, sometimes with the tail perpendicular or above the back. |
|  | Snapping | To snap teeth into the air, noisily. |
| Reaching for the food  (test condition only) | …. | The partner scratches the front fence of the enclosure or puts the paw through the fence towards the tray |

S2 Table: Ethogram of the behaviours coded for test and control sessions.
